# Sensitivity and Specificity of the Unité Rhumatologique Des Affections De La Main (URAM) Scale for Dupuytren Contracture: A Systematic Review and Meta-Analyses

**DOI:** 10.7759/cureus.21636

**Published:** 2022-01-26

**Authors:** Rafael Sanjuan-Cervero, Diego Gomez-Herrero, Pedro Vazquez-Ferreiro, Aurora Sanjuan-Arago, Jaime E Poquet-Jornet, Javier Carrer-Hueso

**Affiliations:** 1 Orthopedics and Traumatology, Hospital de Denia, Denia, ESP; 2 Pharmacy, Hospital Vithas Valencia 9 de Octubre, Valencia, ESP; 3 Ophthalmology, Hospital Virxen da Xunqueira, Cee, ESP; 4 Pharmacy, Hospital de Denia, Denia, ESP; 5 Pharmacy, Hospital Universitario de La Plana, Villareal, ESP

**Keywords:** meta-analyses, specificity, sensitivity, uram, roc curve, dupuytren contracture

## Abstract

Background: Unité Rhumatologique des Affections de la Main (URAM) is a novel and disease-specific questionnaire for Dupuytren contracture, a fibroproliferative disease that affects hands causing progressive contracture in flexion of the fingers.

Objectives: To evaluate the sensitivity and specificity of the URAM scale in Dupuytren contracture.

Materials and Methods: We performed meta-analyses of 10 articles published in PubMed, Embase, Cochrane, Google Scholar, Latin American and Caribbean Health Sciences Literature (LILACS), and in various grey literature databases that describe the use of the URAM and Tubiana scales to assess treatment outcomes in Dupuytren contracture. We built three models: a hierarchical summary receiver operating characteristic (HSROC) model to determine the optimal threshold for defining Dupuytren contracture, a difference in means model to assess the magnitude of the effect of different treatment modalities, and a meta-regression model to determine the effect on patient quality of life questionnaires such as the URAM, according to variations in Tubiana scores after treatment.

Results: The HSROC and bivariate models showed a sensitivity of 80.23% (95% CI: 75.66 to 84.14) and an overall specificity of 2.61% (95% CI: 1.11 to 6.05). The second model showed an overall difference in means of 1.95 (95% CI: -2.86 to -1.04) for partial fasciectomy and collagenase* Clostridium histolyticum *(CCH) injections, and -1.30 (95% CI: -1.77 to -0.83) for partial fasciectomy, and -2.75 (95% CI: -4.73 to -0.78) for CCH. The coefficient obtained in the meta-regression model was -1.666 (95% CI: -4.183 to 0.851).

Conclusion: The URAM scale is highly sensitive to changes in Dupuytren contracture but has low specificity. It also showed a strong correlation with worsening of finger contracture as measured by the Tubiana scale.

## Introduction and background

Dupuytren contracture (DC) is a fibroproliferative disease of the palmar fascia that can affect one or both hands. It causes progressive digital contracture that prevents patients from straightening their fingers, affecting hand function, the performance of basic activities of daily living, and quality of life [1].

Treatments include surgical procedures, such as fasciotomy, partial fasciectomy (FSC), dermofasciectomy, and minimally invasive procedures such as collagenase *Clostridium histolyticum* (CCH) injections, and needle aponeurotomy (NA) [2]. None of these treatments is curative and therefore many patients develop recurrent disease and require repeat treatment [3]. Much research has been done on treatment outcomes in DC, with studies evaluating functional outcomes, patient satisfaction, and perceived quality of life using physical measures and self-report questionnaires [4]. Patient satisfaction with treatment or with treatment outcomes is a multidimensional concept that can be difficult to define and measure and it also depends on aspects related to healthcare structure and delivery [5]. In addition, the studies published to date have used different methods and asked different questions. Some authors have recommended using the Unité Rhumatologique des Affections de la Main (URAM) scale to monitor disease progression and treatment success and enable comparisons between different treatment modalities in DC [6].

The URAM scale was specifically designed to evaluate hand function in DC by the Rheumatology Department at Hospital Lariboisere in Paris, France in 2011. It is the first patient-reported functional outcome measurement tool validated for exclusive use in DC and has been shown to have adequate psychometric properties [7]. It consists of nine multiple-choice questions, meaning it is sufficiently short and easy to use in both daily practice and clinical trials [8,9]. It assesses patients’ perceived ability to perform a range of activities, including activities of daily living, and also addresses symptoms such as stiffness and loss of strength [10]. Several studies have evaluated the reliability (good to excellent) and responsiveness of the scale [11], which in addition has been adapted and validated for use in different languages [7].

The aim of this study was to perform a systematic review and meta-analyses of the sensitivity and specificity of the URAM scale in terms of its ability to define the quality of life in patients with DC and its responsiveness to changes in disease severity following treatment.

This article was previously posted to the Research Square preprint server on June 2, 2020, and to ResearchGate on May 2, 2020.

## Review

Methods

The systematic review was performed according to the recommendations of Eden et al. [12] on review methods, data sources, and search strategies. We addressed two review questions: (1) Using the Tubiana scale as a reference test, how sensitive and specific is the URAM scale for defining quality of life in patients with DC? (2) How sensitive to change is the URAM scale after treatment with FSC and CCH?

We performed a systematic search of PubMed, Embase, Cochrane, Google Scholar, Latin American and Caribbean Health Sciences Literature (LILAC), and Web of Science for articles published between January 1, 1990, and June 1, 2019. The search criteria used in all the databases were combinations of the terms “Unité Rhumatologique des Affections de la Main”, “URAM”, "Dupuytren Contracture", and “Dupuytren”.

Two reviewers (PVF and DGH) independently searched the databases and reviewed the articles retrieved. They also hand-searched the reference lists of relevant articles and reviewed the grey literature to identify clinical trial reports and conference proceedings. Clinical trials, cohort studies, and case-control studies that had used the URAM scale to evaluate DC were included. Authors were contacted when specific information on the use of this scale was missing. To minimize publication bias, no language constraints were placed.

Study Selection

Two researchers (PVF and DGH) independently screened the titles and abstracts to identify suitable texts, which they then reviewed in depth. Disagreements were resolved by a third author (FJCH) only when there was a discrepancy in reporting and reviewing by the first two authors in retrieval, risk of bias assessment.

Data Extraction and Risk of Bias Assessment

Working separately, PVF and DGH transferred all relevant data from the selected articles into standardized forms. The reliability of the entries was checked by another researcher (JEPJ). In addition to effect variables i.e., mean and standard deviation (SD) pre-and post-intervention URAM and Tubiana scores, the data recorded included demographic variables (age, gender, and hand and radius affected) and variables for the stratification analyses in the meta-analyses (e.g., quality, language, study type).

As the studies included in the meta-analyses differed in type, their quality was assessed using the Strengthening the Reporting of Observational Studies in Epidemiology (STROBE) checklist [13] applied separately by two researchers for each article. To minimize bias, a score of 15 or higher was used to identify high-quality studies. Discrepancies i.e., differences in scores that placed a given study above or below the cutoff of 15, were resolved by a third researcher (RSC).

Statistics

Three meta-analyses models were used to answer the research questions: a hierarchical summary receiver operating characteristic (HSROC) model, a difference in means model for pre- and post-treatment URAM scores, and a meta-regression model adjusted for time since treatment.

For the HSROC model, tables summarizing Tubiana and URAM scores reported in each of the studies were created. In both cases, it was assumed that the scores were normally distributed. The data were then presented in 2×2 contingency tables with the URAM scale as the index test and the Tubiana scale as the reference test. The respective thresholds used were 2.5 and 1. The prevalence of DC was established at 100%. In other words, it was assumed that there were no true negatives, meaning all the negative results for the reference test were false negatives. Enabling continuity correction, we then built a hierarchical multinomial regression HSROC model [14], which converts the distribution of the two variables, allowing calculation of the overall receiver operating characteristic (ROC) curve under the assumption that there is an underlying curve for each of the studies included. Each curve is determined by two parameters, α and β, which denote accuracy and asymmetry, respectively. Using these parameters and a θ parameter to denote the positivity threshold, distribution tables were generated for each study assuming that while the distribution of parameters would vary between studies, it would be normal and random (random-effects model). We then estimated the overall ROC curve together with the optimal threshold and corresponding confidence interval. The bivariate model was applied to directly model specificity and sensitivity based on the assumption that the Napierian logarithm of the odds ratio had a normal bivariate distribution in the different studies analyzed [15].

For the second model, standardized mean differences in pre- and post-treatment URAM scores were computed using Cohen’s D and appropriate weighting. The most conservative model was selected in each case [16]. Differences of over 10% were considered to be clinically significant and the results were stratified by type of intervention (FSC or CCH). Each group was finally assigned an overall value.

For the meta-regression model, the dependent variable was changed in URAM scores after treatment (differences in means before and after FSC or CCH) and the independent variables were Tubiana scores, time since treatment, type of treatment, age, and sex. The model with the greatest explanatory power was selected.

Heterogeneity between studies was investigated using the I2 statistic, with high heterogeneity defined as a value of over 50% [16]. Potential sources of heterogeneity were investigated by subgroup analyses (study setting, language, ethnic origin), and the effect of outliers was analyzed in a sensitivity analysis in which studies were excluded one by one.

Analyses were conducted using the metan, metacum, metafunnel, and metandi features in Stata version 15 (StataCorp, College Station, Texas). Differences in means were considered to be significant when the confidence intervals did not cross 0 and clinically significant when there was a difference of at least 10%. Publication bias was assessed using funnel plots and the Begg-Mazumdar test [17].

Results

**Figure 1:**
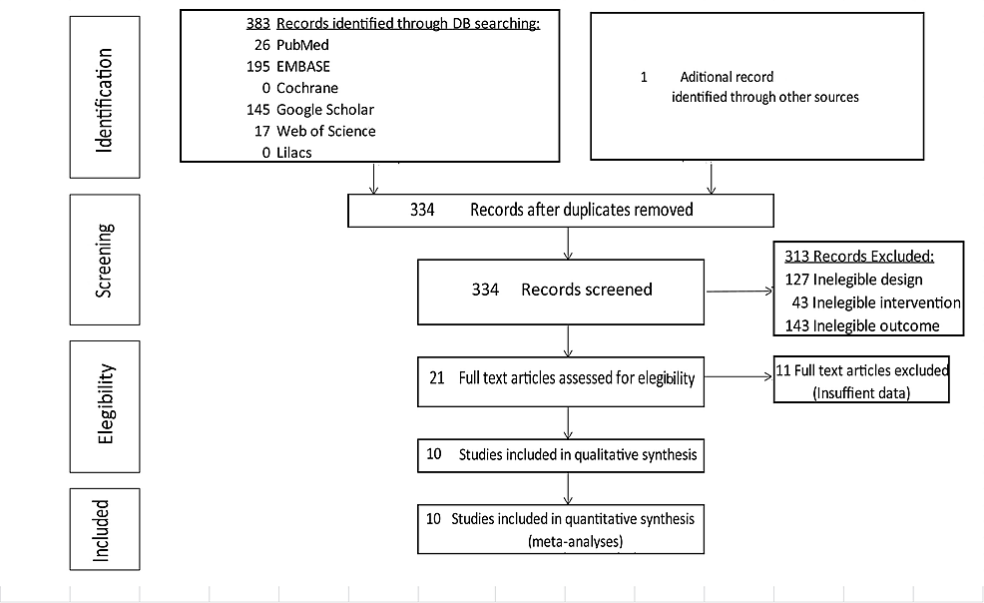
Flowchart of study inclusion DB: Database

Our search strategy retrieved 384 articles (Figure 1) but 50 of these were excluded due to duplication. After screening the titles and abstracts of the remaining 334 articles, 313 were excluded (inadequate study design, missing data, different definitions of disease or disease severity, and publication in a language that could not be translated). Of the 21 articles selected for full-text review, 11 were excluded as they did not contain the information needed for our calculations (URAM scores, Tubiana scores, or degrees of contracture). Ten articles thus were included in the meta-analyses. Eight were used in the HSROC model, nine in the difference in means model, and 10 in the meta-regression model. The main characteristics of the studies are shown in Table 1.

**Table 1:** Characteristics of the studies included in the meta-analyses Study: Study author; Year: Date of publication; N: Number of patients; Country: Country in which study was performed; Type: Study design, CT: Clinical trial, CH: Cohort study; Age: Mean age of patients; Male: Percentage of male patients; Fasciotomy: Percentage of patients who underwent fasciotomy; NA: Needle aponeurotomy; CCH: Percentage of patients who received collagenase *Clostridium histolyticum* injections; Hand: Percentage of right hands operated on; R5: Percentage of interventions involving the fifth radius; R4: Percentage of interventions involving the fourth radius; R3: Percentage of interventions involving the third radius; R2:Percentage of interventions involving the second radius; R1:Percentage of interventions involving the first radius.

Study	Year	N	Country	Type	Language	Age	Male	NA	CCH	Hand	R5	R4	R3	R2	R1
Beaudreuil [7]	2011	53	France	CH	English	63.2	83.02	0	100	54.72	N.A	N.A	N.A	N.A	N.A
Scherman [18]	2013	93	Sweden	CH	English	67	86.75	50	50	53.49	55.81	37.21	6.98	0	0
Warwick [19]	2013	254	International	CH	English	60	87.79	0	100	75.59	75.20	68.90	29.53	09.06	2.36
Bernabé [10]	2014	83	France	CH	English	63	83.02	0	0	N.A	N.A	N.A	N.A	N.A	N.A
Stromberg [8]	2016	140	Sweden	CT	English	67.5	85.26	51	49	58.57	51.43	35.00	06.43	0	0
Broekstra [20]	2017	233	Holland	CH	English	65.5	65.35	0	0	N.A	N.A	N.A	N.A	N.A	N.A
Harrison [21]	2017	71	UK	CH	English	65.7	76.06	54	46	80.28	57.75	12.68	29.58	0	0
Stromberg [22]	2018	152	Sweden	CT	English	66.5	85.27	50	50	58.34	51.28	41.67	07.05	N.A	N.A.
Ferrari [23]	2018	30	France	CH	English	72	83.34	100	0	N.A	N.A	N.A	N.A	N.A	N.A
Wiseman [24]	2018	136	Australia	CH	English	66	80.15	0	100	75.18	N.A	N.A	N.A	N.A	N.A

**Figure 2:**
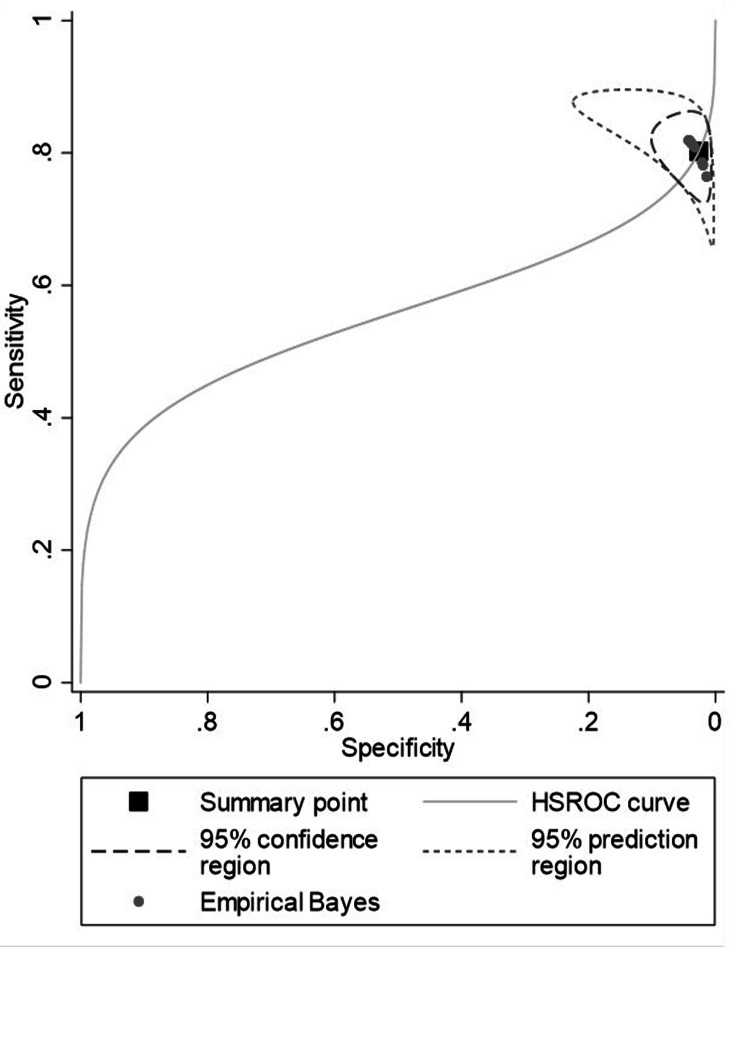
The HSCROC curve with 95% confidence region and empirical Bayes estimate and 95% prediction region HSROC: Hierarchical summary receiver operating characteristic

Application of the HSROC [14] and bivariate models showed an overall sensitivity of 80.23% (95% CI: 75.66 to 84.14) and specificity of 2.61%, (95% CI: 1.11 to 6.05). The diagnostic odds ratio was 0.109, (95% CI: 0.041 to 0.292), with a positive predictive value of 0.824 and a negative predictive value of 7.546 (Table 2). The reference value together with its 95% prediction region is shown in Figure 2.

**Table 2:** Parameters computed for the HSROC and bivariate models Covariance between estimates of E(logitSe) & E(logitSp) 0,0174904; Log likelihood -32,050571 E(logitSe): Logit-transformed sensitivity; E(logitSP): Logit-transformed specificity; Var(logitSe): Variance of logit-transformed sensitivity; Var(logitSp): Variance of logit-transformed specificity; Corr(logits): Correlation; Lambda: Mean accuracy parameter; Theta: Positivity parameter; Beta: Asymmetry parameter; s2alpha: Variance of alpha parameter, s2theta: Variance of theta parameter; Se: Sensitivity; Sp: Specificity; DOR: Diagnostic odds ratio; LR+ and -: Positive and negative likelihood ratios; 1/LR-: Inverse of the negative likelihood ratio; HSROC: Hierarchical summary receiver operating characteristic

Model	Parameter	Coef.	Std. Err.	z	P>z	95% Conf. Interval	
Bivariate	E(logitSe)	1.401	0.136			1.134	1.669
	E(logitSp)	-3.616	0.446			-4.490	-2.742
	Var(logitSe)	0.036	0.051			0.002	0.577
	Var(logitSp)	0.350	0.652			0.009	13.436
	Corr(logits)	1.000	-			-	-
HSROC	Lambda	0.428	2.376			-4.228	5.085
	Theta	2.261	0.160			1.948	2.574
	Beta	1.138	1.034	1.100	0.271	-0.888	3.164
	s2alpha	0.449	0.580			0.036	5.631
	s2theta	0.000	-			-	-
Summary pt.	Se	0.802	0.022			0.757	0.841
	Sp	0.026	0.011			0.011	0.061
	DOR	0.109	0.055			0.041	0.292
	LR+	0.824	0.027			0.773	0.878
	LR-	7.546	3.601			2.961	19.228
	1/LR-	0.133	0.063			0.052	0.338

**Figure 3:**
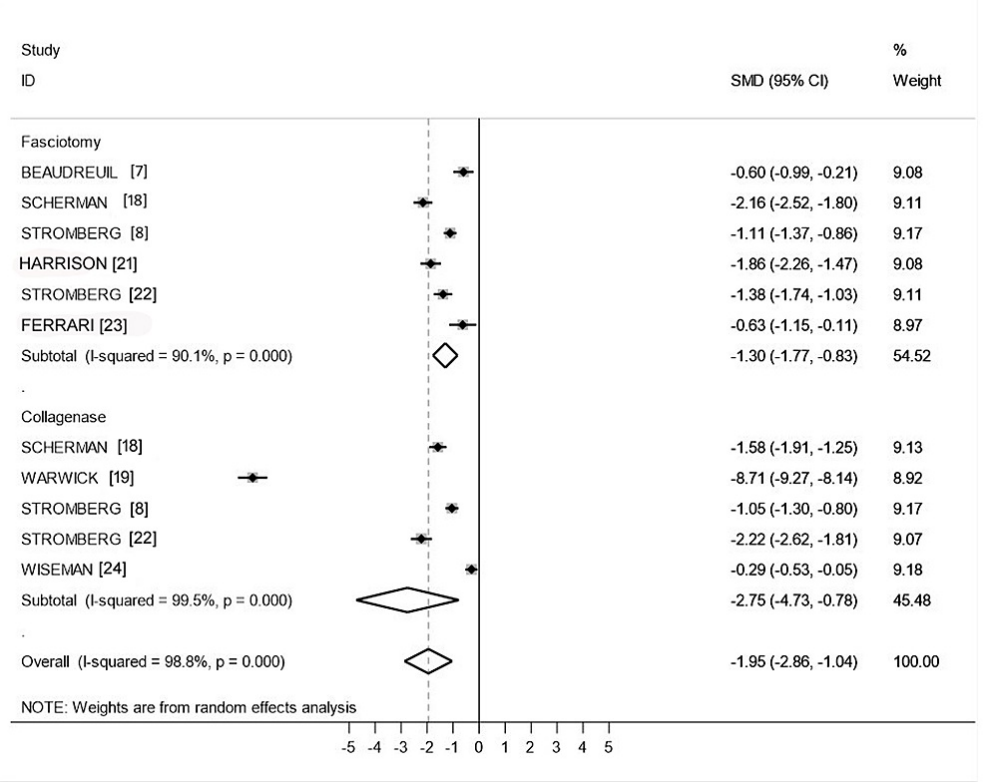
Meta-analyses of differences in URAM scores according to procedure URAM: Unité Rhumatologique des Affections de la Main

In the differences in mean model, we obtained a value of -1.30 (95% CI: -1.77 to -0.83) for FSC and -2.75 (95% CI: -4.73 to -0.78) for CCH. The overall value was -1.95 (95% CI: -2.86 to -1.04) (Figure 3).

The only significant variable in the meta-regression analysis of the influence of variations in Tubiana scores on URAM scores was the time between treatment and completion of the URAM questionnaire (Table 3). The coefficient for this model was 1.666 (95% CI: -4.183 to 0.851).

**Table 3:** Meta-regression Random-effects model estimate of between-study variance: Tau2 = 3.18 % residual variation due to heterogeneity: I2 = 75.40% Proportion of between-study variance explained: R2 = 28.85% Tubiana: Mean difference in Tubiana score; Time: Time from treatment to measurement of final outcome; Constant: Constant of the model

	Coef.	Std. Err.	t	P > |t|	95% Conf. Interval	
Tubiana	-1.666	0.979	-1.70	0.150	-4.183	0.851
Time	0.014	0.009	1.55	0.182	-0.009	0.037
Constant	-13.392	2.713	-4.94	0.004	-20.366	-6.417

Discussion

Our meta-analyses showed high sensitivity (80.2%, 95% CI: 75.7% to 84.1%) and low specificity (2.6%, 95% CI: 1.11% to 6.1%) at the optimal threshold for the URAM scale as a diagnostic test for DC. The scale was also very sensitive to change after treatment with FSC and CCH, with an overall difference in means of -1.95 (95% CI: -2.86 to -1.04). It was more responsive to changes after CCH. The scale was also able to capture the effect of time since treatment, although to a lesser extent (nonsignificant coefficient). The high sensitivity observed is to be expected as the URAM scale contains several questions on hand mobility. It is also consistent with the strong correlation observed between URAM and Tubiana scores in previous validations [7] and with high sensitivity values reported for the Disabilities of the Arm, Shoulder and Hand (DASH) (82%) and QuickDASH (79%) questionnaires, although in these cases, meaningful change was measured using a very different methodology [25]. The low diagnostic specificity observed (2.6%) is much lower than the rate reported for DASH (overall specificity, 74%) [25], although to our knowledge, the diagnostic accuracy of DASH has not yet been analyzed in the specific setting of DC, but rather in studies analyzing different diseases [26] or responsiveness to changes after corrective surgery for DC [27]. Its diagnostic specificity for DC thus is unknown and is probably much lower than rates reported for general upper arm disabilities. Notwithstanding, the URAM scale has been reported to outperform other scales in terms of its specificity for DC [28]. Although we do not have data to confirm this superior performance, we did find a relatively good balance between sensitivity and specificity (around 60%) in the underlying ROC curve (as seen above in Figure 2), suggesting good disease-specific performance in DC.

The theoretical advantage that the URAM scale offers over other scales is that it was designed to measure functional outcomes in DC, although its usefulness for assessing overall treatment outcomes has been questioned as it does not address aspects such as pain or cosmetic problems [27].

The interpretability of the URAM scale can be assessed based on clinical relevance and the minimal clinically important difference (MCID), which are measures of variations in scores over time that are perceived as important by patients or that reveal significant differences between patients. The clinical relevance of URAM score changes was demonstrated in the original study describing the development and validation of this tool through the observation of a significant correlation between variations in URAM and Tubiana scores after treatment [7]. The MCID refers to the smallest change in status that patients consider important. Estimated MCID values for the URAM scale in the literature are 2.9 points for patients treated with NA [7] and 10.5 points for those treated with open surgery (FSC or dermofasciectomy) [27]. The results of our meta-regression analysis are consistent with these results, as we detected an improvement of 1.67 points in the URAM scale for each change in the Tubiana stage (improvement of approximately 45º). We are unable to compare our results with those of other scales such as DASH and QuickDASH, both validated for measuring outcomes in DC [27], as none of the studies analyzed detected MCIDs that reached the “official” clinically important change [29] or other independently established thresholds [25]. As indicated by a growing number of authors, thus, DASH, which is the most widely used tool for measuring treatment outcomes in DC, may not be the most useful tool for this purpose [27].

The low specificity observed for the URAM scale in our meta-analyses has several explanations. On the one hand, our model was based on a series of assumptions, including the absence of false positives (perfect specificity) and the thresholds used to define DC. There is currently no agreement on where the line between disease and recurrence lies, although some progress is being made [21]. On the other hand, the URAM scale was specifically designed for DC, but it could theoretically be used in other diseases such as carpal tunnel syndrome, as it addresses hand mobility problems that are not specific to DC. Question 9, for example, evaluates problems with pinch, which is generally a greater problem in patients with carpal tunnel syndrome than in those with DC, who face more difficulties straightening their fingers. Additional sources of heterogeneity in our model are the diverse criteria used to measure contracture [30], the different time points at which treatment outcomes were measured, and even doubts about the applicability of the scale in different languages and cultures, although high consistency has been reported for the validated versions of the URAM scale in several languages.

The limitations of this study are linked to the varying degrees of contracture severity in the samples analyzed, potential selection bias, and potential information bias as the URAM scale was not designed as a diagnostic test.

## Conclusions

In conclusion, our meta-analyses show that the URAM scale has high sensitivity and low specificity for DC, although it was sensitive to clinically significant changes following treatment.
